# Eating Behavior and Nutritional Profile of Children with Autism Spectrum Disorder in a Reference Center in the Amazon

**DOI:** 10.3390/nu16030452

**Published:** 2024-02-04

**Authors:** Rayanne Vieira da Silva, Daniela Lopes Gomes

**Affiliations:** Nucleus of Behavior Theory Research, Federal University of Pará, Belém 66087-110, Brazil; rayanne.vs@hotmail.com

**Keywords:** eating behavior, nutritional status, autism spectrum disorder, body mass index

## Abstract

There is no single pattern for the evolution of the nutritional status of children with autism spectrum disorder (ASD). Previous studies have found a tendency towards food selectivity with food monotony and difficulties with food texture in children with ASD, but studies in this area, especially in Brazil, are still scarce. The nutritional profile and changes in eating behavior were analyzed in patients with autism spectrum disorder assisted at a reference center in Belém. Eating behavior was assessed using the Labyrinth Scale, nutritional status assessment through weight and height (to calculate body mass index—BMI), and consumption food through the 24 h reminder. A total of 80 children of both sexes participated in the study, the majority of whom were male (80%), 47.5% eutrophic, while for the food consumption of the children evaluated, there was an average energy consumption of 1911 kcal daily, with 57.3%, 15.4%, and 27.3% of carbohydrates, proteins, and lipids, respectively. In relation to eating behavior, the highest averages were demonstrated in the domains of food selectivity, behavioral aspects, and mealtime skills. Masticatory motor scores showed a positive correlation with weight, BMI, and the amount of energy consumed by the child. The gastrointestinal symptoms score showed a negative correlation with the child’s age. Regarding mealtime skills, a negative correlation was observed with the proportion of carbohydrates in the diet and a positive correlation with the proportion of lipids consumed in the children’s diet. Therefore, knowing the main changes in eating behavior is important to ensure a complete and safe approach for each patient.

## 1. Introduction

Autism or Autism Spectrum Disorder (ASD) is, by definition, a neurodevelopmental disorder characterized by atypical development, behavioral manifestations, deficits in communication and social interaction, and repetitive and stereotyped behavior patterns, and may present a restricted repertoire of interests and activities [1]. According to the World Health Organization (WHO) [2], this syndrome can be diagnosed before the age of three. Its etiology is still unknown, although multifactorial aspects of its determination originating from environmental, genetic [3], and epigenetic [4] variables are currently known. Examples of factors currently studied as being correlated with the condition are parents’ age and race, type of birth, low birth weight, and gestational age at birth, among other aspects [5].

The diagnosis of autism is established using behavioral criteria, and, currently, professionals in most countries use the criteria described in the *Diagnostic and Statistical Manual of Mental Disorders* (DSM-V) [6]. According to these criteria, the child must present at least six symptoms from a list of twelve symptoms presented by the DSM-V, considering that at least two of the symptoms must be in the area of social interaction and at least one in the area of behaviors restricted, repetitive, and stereotyped.

It is very common for autistic children to have nutritional deficiencies, as the majority have a monotonous diet, due to factors such as food selectivity and food neophobia. Therefore, data suggest that autistic children are two to three times more likely to be obese than the neurotypical population [7].

A central aspect of selective eating is food neophobia, characterized by the propensity to refuse to try new foods, and is prevalent in early childhood development, even in neurotypical children. Food neophobia is intensified when associated with ASD, being present for prolonged periods from childhood to youth. The progression of this disorder is directly associated with the level of severity of the cognition, social, and communicative impairment of the autistic patient [8].

Furthermore, this disorder is also related to a limitation in dietary variety, with adverse impacts especially associated with reduced consumption of nutrient-rich foods, such as fruits and vegetables, and increased consumption of inappropriate foods, favoring the additive risk of poor nutrition [8], contributing to people with autism presenting an increase in body mass index (BMI). Souza et al. [9] states that there is no single pattern for the evolution of the nutritional status of children with ASD, as most studies have a very small number of participants. Grokoski [10] and Vitória [11] demonstrated a high prevalence of obesity, but other research also indicates a prevalence of normal weight in this population, as in the study of Mari-Bauset et al. [12].

Furthermore, we highlight that the nutritional status is directly related to children’s eating behavior; therefore, if food preferences are for sweets and ultra-processed foods consumed in large quantities, the nutritional status can evolve into overweight, but if the child has selectivity severe food intake with less caloric foods and in small quantities, the nutritional status can progress to low weight. Rocha et al. [13] found a tendency towards food selectivity with a predominance of repetition of the same foods and difficulties with texture in children with ASD, but studies in this area, especially in Brazil, are still scarce, especially in children undergoing therapeutic intervention in reference centers.

Evidence from animal models suggest that microbial shifts in the gut may produce changes with the clinical profile of autism, with proposed mechanisms including production of toxins due to intestinal dysbiosis and immunological and metabolic changes [14,15]. For this reason, it is recommended that the study of nutritional status be associated with the study of eating behavior, in particular, studies that assess the presence of food selectivity. Perhaps more than any other behavioral disorder in children and adolescents, early detection of eating disorders is essential. Given this, increasing evidence has indicated that the earlier the therapeutic interventions, the better the long-term prognosis [16].

The present study aimed to analyze the nutritional profile and changes in the eating behavior of children with autism spectrum disorder assisted by the Integrated Center for Inclusion and Rehabilitation—CIIR in Belém.

## 2. Materials and Methods

### 2.1. Type of Study

This is a cross-sectional, descriptive, and analytical study, with the target audience being children with ASD and their respective caregivers assisted by the Autistic Spectrum Disorder Care Center (NATEA), located at the Integrated Center for Inclusion and Rehabilitation (CIIR) in Belém-PA, from June to August 2022, on Fridays, in the morning and afternoon shifts.

The CIIR is a public institution created to serve people with disabilities throughout the state of Pará, with the Autistic Spectrum Disorder Assistance Center (NATEA) being a reference in the North of Brazil in the care of people with autism, providing a range of therapies to enhance the rehabilitation of users, which belongs to the Integrated Center for Inclusion and Rehabilitation (CIIR).

### 2.2. Ethical Aspects

The present study was approved by the Ethics and Research Committee of the Tropical Medicine Center—NMT of the Federal University of Pará—UFPA, respecting the Norms for Research Involving Human Beings (Resolution no. 466/2012) of the National Health Council, under the opinion number 5.354.653, in April 2022. All of the children’s primary caregivers agreed to participate in the research and signed the Informed Consent Form (ICF).

### 2.3. Participants

A total of 80 children and their respective parents/caregivers participated in this study. The inclusion criteria adopted were the child must have been diagnosed with ASD; be between 3 years old and 11 years and 11 months old upon signing the Informed Consent Form (ICF); receive services at the CIIR; be a CIIR attendee; and their caregiver agreed to participate in the research by signing the ICF. And the exclusion criteria were not having a closed diagnosis of ASD; being less than 3 years old or over 12 years old; being a twin; not attending CIIR; having caregivers who did not agree to participate in the study; and having parents or guardians present at the time of the consultation who did not actively participate in the child’s care.

Initially, caregivers were approached in the NATEA waiting room while they waited for the therapies to begin. At this point, the reasons that led to this research and its benefits for the participants were clarified, as well as an invitation to participate in the study. Through an interview completed in full by legal guardians, after accepting to participate in the research and after reading and signing the TCLE and TALE, the caregivers and children began their participation in the study.

Caregivers were informed that they could choose not to approve the child’s participation in the research and that they would have the right to withdraw from participation at any time at no cost for the child’s treatment in the center. Furthermore, it was guaranteed that the information will be kept confidential.

### 2.4. Instruments

The Eating Behavior Assessment was carried out in patients with ASD using the Labyrinth Scale validated in Brazil by Lázaro et al. [17] to evaluate dietary changes in these children. This is a specific scale for patients with ASD, which can be applied at any age, with the person responsible for the child responding. The scale is divided into the following dimensions: chewing motor skills, food selectivity, behavioral aspects, gastrointestinal symptoms, sensory sensitivity, and mealtime skills. The dimension of food selectivity is emphasized in this study. For each question, the participant must choose the option that best applies to their own case, with the alternatives being “never”, “rarely”, “sometimes”, “often”, or “always”. The answers have a score from 1 to 5, with “never” equivalent to 1 point and “always” equivalent to 5 points; that is, the more “always” answers, the higher the score and the more food selectivity the patient presents.

Then, data were collected to assess the children’s nutritional status, where weight was collected with a digital scale, with a variation of 0.1 kg and capacity of up to 150 kg, and height with an aluminum anthropometer with 0.1 cm precision of the Welmy brand (certified by the National Institute of Metrology, Quality and Technology—INMETRO and recently calibrated) provided by the researcher. The children were weighed and measured with clothes on and without shoes. The Body Mass Index (BMI) was calculated using the formula of weight (W) over height (H) squared (W/H2). The children had their anthropometric assessment carried out in accordance with the instructions of the Food and Nutrition Surveillance System (SISVAN) [18].

The BMI Growth Charts for Age, for females and males, from the World Health Organization [19,20] were used to classify children as underweight for their age, eutrophic, overweight, or obese.

To assess food consumption, three 24 h recalls (R24h) were applied on alternate days to establish the average energy and macronutrient intake of each child. The R24h were answered by the child’s guardian. Among the recalls applied, two recalls were carried out on weekdays and one on the weekend, as in the latter, eating habits tend to be different from the weekly pattern and must be included to estimate usual consumption. The first was carried out in person and the other two by telephone [21].

In all R24h, they were first asked how many meals were eaten, then the foods and quantities of each in household measurements. The analysis of these recalls was carried out using the Webdiet^®^ software, v. 4.0, analyzing the average of the 3 recalls and estimating the average energy consumed in calories (Kcal) and the proportion of macronutrients in percentage (%).

### 2.5. Data Analysis

For statistical analysis, SPSS software, version 25.0, was used. The results for categorical variables were expressed as absolute frequency and proportion, and for continuous variables, they were expressed as mean and standard deviation. The Pearson correlation test was applied to evaluate bivariate correlations, and the *t*-test for independent samples was applied to compare outcomes between groups. For all analyses, a statistical significance level of *p* < 0.05 was considered.

## 3. Results

The total number of participants who met the inclusion criteria was 100 participants, of which authorization was granted for 84 participants; however, there were two pairs of twin brothers who were excluded from the sample.

Eighty children with ASD participated in the research, with a mean age of 6.9 ± 2.5 years, the majority of whom were male (80%). It was found that 47.5% (*n* = 38) of the children were eutrophic and 35% (*n* = 28) were obese (Table 1).

**Figure 1:**
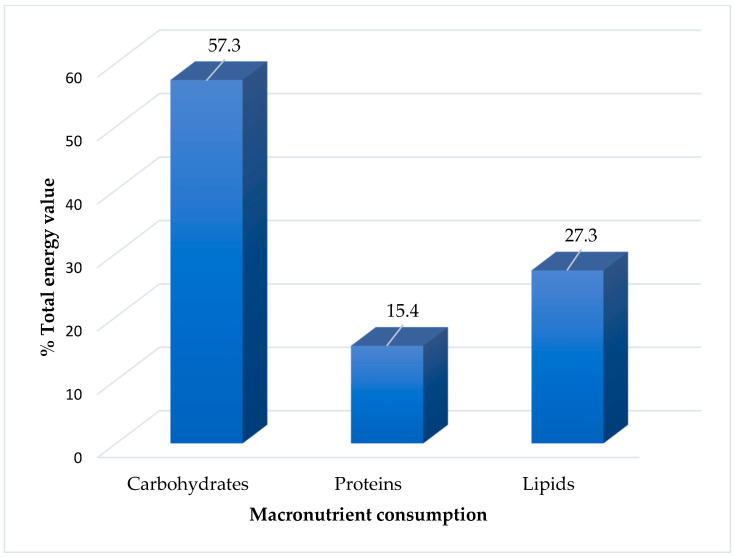
Consumption of macronutrients from the usual diet of children with autism spectrum disorder followed in a public reference center in the Amazon.

There was an average energy consumption of 1911 kcal per day and an average macronutrient consumption of 57.3% carbohydrates, 15.4% proteins, and 27.3% lipids (Figure 1).

Table 2 presents the description of children’s eating behavior in each of the six domains of the Labyrinth Scale, demonstrating that the highest means were observed in the domains of food selectivity (38.3 ± 14.1), behavioral aspects (25.0 ± 9.9), chewing motor skills (22.1 ± 11.0), and mealtime skills (8.5 ± 4.3), according to the scores for each domain of the scale.

**Figure 2:**
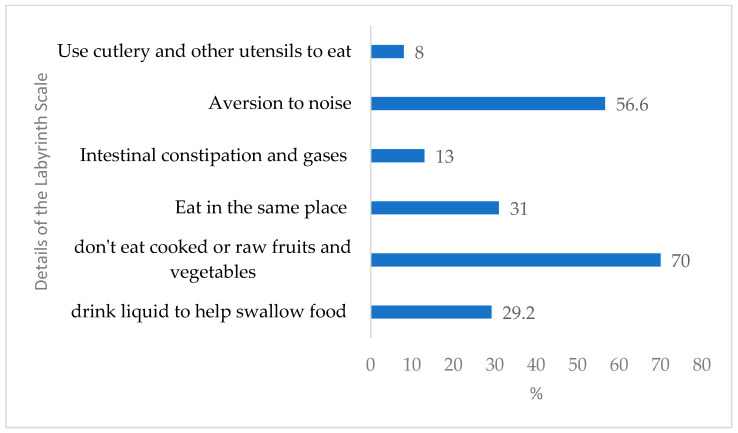
Details of the questions of the Labyrinth Scale of children with autism spectrum disorder followed in a public reference center in the Amazon.

Regarding each domain, in “chewing motor skills”, the most frequent behavior was the child needing to drink liquid to help swallow food (29.2%). Within the food selectivity domain, 70% of children avoided eating cooked and/or raw fruits and vegetables. Regarding behavioral aspects, always eating in the same place had the highest percentage (31%). In the domain relating to gastrointestinal symptoms, constipation, dry and constipated intestines, constipation, gas, and bloating in the belly were presented by 13% of children, while in the domain of sensory sensitivity, being bothered by noises was the most frequent behavior (56.6%). Finally, regarding mealtime skills, difficulties in using cutlery and other utensils was the least common behavior shown by children (8%) (Figure 2).

Table 3 shows the nutritional factors correlated with the domains of the Labyrinth Scale. It is possible to verify that the masticatory motor score showed a positive correlation with weight (r^2^ = 0.359; *p*-value = 0.001), BMI (r^2^ = 0.489; *p*-value = 0.000), and the amount of energy consumed by the child (r^2^ = 0.379; *p*-value = 0.000). The gastrointestinal symptoms score showed a negative correlation with the child’s age (r^2^ = 0.267; *p*-value = 0.008). For the meal skills score, a negative correlation was observed with the proportion of carbohydrates in the diet (r^2^ = −0.240; *p*-value = 0.016) and a positive correlation with the proportion of lipids consumed in the children’s diet (r^2^ = 0.193; *p*-value = 0.043).

## 4. Discussion

It was observed that the majority of the sample was made up of male children, a result similar to those found in studies carried out by De Paula et al. [22] and Marques et al. [23], who verified the prevalence of males in the diagnosis of ASD. The results corroborate the ratio between the male and female sexes of 4:1, according to DSM-5 [6], and the ratio of 3:1, according to the meta-analysis carried out by Loomes et al. [24].

Most of the children evaluated in this study presented a eutrophic nutritional status, followed by obesity. It is known that the nutritional status of children with ASD is influenced by inadequate food consumption and factors related to eating behavior [25]. However, Souza et al. [9] highlights that the results of some studies are conflicting regarding the nutritional status of these patients, as reports in the literature are often based on a small number of participants and different measurement methods, which makes data congruence difficult.

According to Melo et al. [26], there are data that suggest that autistic children are two to three times more likely to be obese than the neurotypical population. In accordance with this statement, studies carried out by Attle et al. [27], Grokoski [10], and Vitória [11] demonstrated a high prevalence of obesity in these individuals. On the other hand, in the present study, a prevalence of eutrophic nutritional status was found, corroborating the data from the research by Mari-Bauset et al. [13].

However, when we observe in the present study a sum of overweight (*n* = 4) and obesity (*n* = 28), we have 40% of children who are overweight, showing that it is common for autistic children to have nutritional deficiencies, as the majority have monotonous eating due to several factors, such as food selectivity and food neophobia [22]. This set of data suggests that variations in nutritional status may indeed be common in this population, with the variation found from low weight to obesity being valid.

According to the present study, the domains of food selectivity, behavioral aspects, and mealtime skills presented higher averages in relation to the six domains of eating behavior on the Labyrinth Scale, in line with what is exposed in the literature, where the act of eating is an experience that involves sensorial varieties because it consists of eating foods with different appearances, smells, textures, and tastes [12].

Atypical eating behavior has been recognized globally in autism since autism was conceptualized as a diagnosis by Kanner [28]. Mayes and Zickgraf [29] observed that 70.4% of people with autism showed atypical eating behavior, while in the study by De Paula et al. [22], this total was 100% of the sample.

Children with ASD may present changes in eating behavior, which, regardless of prevalence, contribute in some way to irregular food consumption. Therefore, such behaviors have a deleterious effect on the child’s development, as they are in a period of growth, both physical and neuropsychomotor development, which depends on adequate and balanced nutrition [17].

Food selectivity is a predominant atypicality in autistic people, although eating behavior has other variants. Rocha et al. [14] demonstrated that there is a tendency towards food selectivity, with a predominance of repeating the same foods and difficulties with texture, justifying the prevalence of this food selectivity. The study by De Paula et al. [22] also shows a higher score in changes in food selectivity in people with ASD.

Moraes et al. [30] observed the significant presence of food selectivity in most participants with ASD, presenting mainly as refusal and a limited food repertoire. This eating behavior may be directly linked to an unusual sensitivity to touch, as well as for other reasons [31]. In the study by Rodrigues [32], this sensory sensitivity was also evidenced in children with ASD.

Within the specificities of food selectivity, there are some sensory characteristics of foods, and autistic people tend to be more vulnerable to characteristics such as odor, texture, color, and temperature. However, some textures become the preference of autistic children [33]. Siddiqi et al. [34] explain that the preference for grains is mainly cereals, although these aspects tend to improve with age.

Sharp et al. [25] showed that eating problems among children with ASD are five times greater than in their peers without ASD, suggesting that food selectivity contributes to nutritional losses in children with ASD. Likewise, our study showed a high score, with an average of 38.1 in relation to the other domains of the scale.

Regarding to the domain of altered behavioral aspects, the behavior of always eating in the same place was the most frequent, and these ritualistic actions interfere with nutrient intake. A survey carried out by Abdallah [35] showed that the nutritional intake of 414 children with ASD with an average age of 9.63 years, who had eating rituals, did not meet the recommended intake of fiber, choline, potassium, and vitamins D and K in the majority of children, compromising child development.

Lázaro [17] argues that the act of eating is learned socially. Therefore, ASD problems regarding socialization make it difficult to eat in a group, which makes learning through imitation more difficult, thus leading to impaired eating behavior, such as not being able to sit at the table for the entire meal and removing food from someone else’s plate, as demonstrated in the present study for changes in the mastery of mealtime skills.

In the present study, there was a significant positive correlation between the masticatory motor score and weight, BMI, and the amount of energy consumed by children. Children with ASD may present changes in eating behavior, which, regardless of prevalence, contribute in some way to irregular food consumption. Therefore, such behaviors have a deleterious effect on the child’s development, as they are in a period of growth [12].

Considering chewing ability, it is known that swallowing encompasses a set of coordinated motor mechanisms. The chewing and swallowing process has voluntary and involuntary phases. Therefore, there are biological and organic factors of the child and, on the other hand, environmental factors (related to family conditions and eating experiences) that can interfere with the masticatory motor process and, consequently, eating [12].

The gastrointestinal symptoms score showed a negative correlation with the child’s age. It is known that gastrointestinal disorders are among the most common medical conditions associated with autism. It is common for constipation, diarrhea, and gastrointestinal reflux, among other conditions, to appear. These gastrointestinal problems can affect people with autism of any age [36], although the present study indicated that symptoms are more frequent in younger children.

Finally, the meal skills score showed a negative correlation with the percentage of carbohydrates and a positive correlation with the amount of lipids consumed in the children’s diet. As the literature indicates, these eating difficulties affect at least 70% to 90% of people with autism, who have incorrect eating habits more regularly than non-autistic children [37].

This fact demands actions aimed at promoting health, such as a healthy eating policy. Therefore, a dietary intervention aims to improve the physical health and well-being of these individuals, and nutritional monitoring with autistic children is essential, contributing to the correction of inadequate eating habits, as well as promoting health and quality of life.

## 5. Conclusions

In this study, it was possible to observe a eutrophic nutritional profile; however, a greater propensity to be overweight was observed. The participants presented greater changes in the domains related to food selectivity, behavioral aspects, and mealtime skills. It was possible to verify that the masticatory motor score showed a positive correlation with the weight and amount of energy consumed by the child. The gastrointestinal symptoms score showed a negative correlation with the child’s age. For the meal skills score, a negative correlation was observed with the proportion of carbohydrates in the diet and a positive correlation with the proportion of lipids consumed in the children’s diet.

This study has some limitations, such as not including samples from children from other treatment centers in Brazil, especially from different regions, and not including other interesting outcomes, such as an assessment of the intestinal microbiome. We suggest that future studies include children from different Brazilian regions and compare public treatment centers with private treatment centers, as well as including other outcomes, such as body composition and intestinal microbiome.

Therefore, children with ASD are part of a nutritional vulnerable group, and knowing the main changes in eating behavior is important to ensure a treatment that is complete, safe, and appropriate for each patient.

## Figures and Tables

**Table 1:** Nutritional profile of children with autism spectrum disorder attending a public reference center in Belém-PA.

Nutritional Status Classification (BMI ^1^/Height)	N = 80	%	*p*-Value ^2^
Thinness	10	12.5	<0.0001
Eutrophic	38	47.5	
Overweight	4	5.0	
Obesity	28	35.0	

^1^ Body Mass Index (BMI); ^2^ Chi-square test.

**Table 2:** Characterization of the eating behavior of children with autism spectrum disorder followed in a public reference center in the Amazon.

Domain	Average ± SD *	Range (Min–Max)	*p*-Value
Masticatory motor skills	22.1 ± 11.0	11–46	<0.001
Food selectivity	38.3 ± 14.1	14–68	
Behavioral aspects	25.0 ± 9.9	11–45	
Gastrointestinal symptoms	11.9 ± 4.4	8–26	
Sensory sensitivity	14.0 ± 5.9	5–29	

* SD = standard deviation.

**Table 3:** Nutritional factors correlated with aspects of eating behavior of children with autism spectrum disorder followed in a public reference center in the Amazon.

Eating Behavior	Age	Weight	BMI ^1^ (kg/m^2^)	Energy	Carbohydrates	Proteins	Lipids
Masticatory motor skills	r^2^ = 0.162	r^2^ = 0.359 *	r^2^ = 0.489 *	r^2^ = 0.379 *	r^2^ = 0.028	r^2^ = −0.139	r^2^ = 0.121
	*p* = 0.076	*p* = 0.001	*p* = 0.000	*p* = 0.000	*p* = 0.404	*p* = 0.109	*p* = 0.142
Food selectivity	r^2^ = 0.112	r^2^ = 0.130	r^2^ = 0.163	r^2^ = 0.060	r^2^ = 0.113	r^2^ = −0.024	r^2^ = −0.057
	*p* = 0.161	*p* = 0.126	*p* = 0.075	*p* = 0.298	*p* = 0.159	*p* = 0.417	*p* = 0.307
Behavioral aspects	r^2^ = −0.028	r^2^ = 0.132	r^2^ = 0.132	r^2^ = 0.107	r^2^ = 0.109	r^2^ = 0.060	r^2^ = −0.178
	*p* = 0.402	*p* = 0.121	*p* = 0.122	*p* = 0.173	*p* = 0.168	*p* = 0.298	*p* = 0.057
Gastrointestinal symptoms	r^2^ = 0.267 *	r^2^ = −0.064	r^2^ = 0.098	r^2^ = 0.149	r^2^ = −0.060	r^2^ = 0.042	r^2^ = −0.005
	*p* = 0.008	*p* = 0.287	*p* = 0.194	*p* = 0.094	*p* = 0.300	*p* = 0.354	*p* = 0.482
Sensory sensitivity	r^2^ = 0.018	r^2^ = 0.164	r^2^ = 0.092	r^2^ = 0.162	r^2^ = −0.058	r^2^ = 0.215 *	r^2^ = −0.123
	*p* = 0.438	*p* = 0.074	*p* = 0.209	*p* = 0.075	*p* = 0.306	*p* = 0.027	*p* = 0.139
Meal skills	r^2^ = −0.075	r^2^ = 0.012	r^2^ = 0.121	r^2^ = 0.141	r^2^ = −0.240 *	r^2^ = 0.074	r^2^ = 0.193 *
	*p* = 0.254	*p* = 0.458	*p* = 0.143	*p* = 0.106	*p* = 0.016	*p* = 0.256	*p* = 0.043

^1^ Body Mass Index (BMI); Pearson correlation test; * Statistical significance.

## Data Availability

The data are not publicly available, as they contain the personal information of the participants involved. Therefore, the data of this work are confidential to maintain the privacy of those involved.
